# Temporal accuracy of human cortico-cortical interactions

**DOI:** 10.1152/jn.00956.2015

**Published:** 2016-02-03

**Authors:** Idan Tal, Moshe Abeles

**Affiliations:** ^1^Gonda Multidisciplinary Brain Research Center, Bar-Ilan University, Ramat-Gan, Israel; and; ^2^The Hebrew University of Jerusalem, Jerusalem, Israel

**Keywords:** MEG, cortico-cortical interactions, temporal precision

## Abstract

The precision in space and time of interactions among multiple cortical sites was evaluated by examining repeating precise spatiotemporal patterns of instances in which cortical currents showed brief amplitude undulations. The amplitudes of the cortical current dipoles were estimated by applying a variant of synthetic aperture magnetometry to magnetoencephalographic (MEG) recordings of subjects tapping to metric auditory rhythms of drum beats. Brief amplitude undulations were detected in the currents by template matching at a rate of 2–3 per second. Their timing was treated as point processes, and precise spatiotemporal patterns were searched for. By randomly teetering these point processes within a time window *W*, we estimated the accuracy of the timing of these brief amplitude undulations and compared the results with those obtained by applying the same analysis to traces composed of random numbers. The results demonstrated that the timing accuracy of patterns was better than 3 ms. Successful classification of two different cognitive processes based on these patterns suggests that at least some of the repeating patterns are specific to a cognitive process.

there is a general consensus that higher brain functions are associated with interactions among several cortical areas. In the late 1980s, a number of researchers (Eckhorn et al. 1988; Gray et al. 1989; Gray and Singer 1989; Mioche and Singer 1989) reported that groups of spatially distributed neurons in the cortex were involved in synchronous oscillatory activity. These findings raised the possibility that the cerebral cortex might utilize the time domain for coding by synchronizing the discharges of neurons with millisecond precision. Synchronized self-generated networks thus could be a coordinating mechanism among several cortical areas (Abeles 1982). Electroencephalographic (EEG) and magnetoencephalographic (MEG) recording methods have provided growing evidence of the correlations between synchronous activity in different frequency bands and a wide variety of cognitive processes such as visual grouping (Vidal et al. 2006), attention (Aftanas and Golocheikine 2001; Jensen et al. 2007; van Ede et al. 2011), memory (Jensen et al. 2007; Osipova et al. 2006), and sensorimotor coordination (Krause et al. 2010; Müller et al. 2000; Pollok et al. 2009; Praamstra et al. 2003). Time-delayed correlations were shown to be specific to voluntary drawing movements (Shmiel et al. 2005, 2006). Measures such as correlation, coherence, and phase synchronization between brain regions are used to search for functionally connected networks and to characterize their dynamics. The time courses of these dynamics range from transient phase-locking of tens of milliseconds induced by simple sensory stimulation, to coherent activity lasting several seconds in a complex cognitive task. All these methods require the analysis of relatively long stretches of data, whereas mental processes such as judging co-occurring shape and color take ∼30 ms per item (Treisman and Sharon 1990). The methods developed in this study allow for almost instantaneous detection of coordination among multiple cortical sites.

Cortico-cortical interactions can be mediated through cortico-cortical connections in the white matter. Almost all these connections are excitatory, whereas local cortical connections are both excitatory and inhibitory, but the majority are associated with excitatory synapses (Braitenberg and Schüz 1991). In other words, when two cortical areas start to interact, they are expected to excite each other, thus increasing the local activity in each. Shortly afterward, inhibition will quench this positive excitatory feedback. Quenching can restore activity to its overall background level or can also result in a brief burst of periodic oscillations. Similarly, specific thalamo-cortical connections are excitatory. When a volley of thalamic input reaches the cortex, a brief increase in activity is typically observed. This increase results in the primary evoked response (ER) observed in the EEG or electrocorticogram (ECoG). Logically, there should be brief amplitude undulations in cortical activity that appear either synchronously or with a fixed delay in several regions. In this report, we term this type of brief undulation a “mini evoked response” (mini ER). This scenario has been found in the cortex of subjects engaged in various mental activities (such as counting down in steps of 7, recalling movie scenes, etc.). Similarly, the distribution of these mini ERs over the cortex was shown to be different in different mental activities (Abeles 2014).

However, in these experiments the mental activities required of the subjects were not fully repetitive. The current study was designed to characterize the temporal coherence of mini ERs over multiple points spread over the cortex. For this purpose it examined entirely repetitive cognitive processes involving the resynchronization of finger taps to changes in drum-beat meter. There are numerous studies on changes in the timing of periodic beats (Michon 1967; Praamstra et al. 2003; Repp 1999; Schulze 1992; Thaut and Kenyon 2003) but, to our knowledge, none in which the meter is randomly changed.

For each point of interest (POI) over the cortical hull, we first detected mini ERs by using a template-matching scheme. We then examined patterns or sequences of mini ER occurrences. These patterns were identified in terms of the intervals between repeating sequences of mini ERs. The precision of timing within these patterns was studied by randomly teetering the event timing, and the specificity to changes in meter was tested. The results show that the patterns were very precise (up to ± 1 ms) and specific to the behavior.

## METHODS

### Behavior

Subjects received payment to participate in the study and signed a consent form approved by the University internal ethics committee. Subjects (*n* = 9, males) were instructed to listen to drum beats with meters of 2 (primary beat followed by a secondary beat) or 3 (primary beat followed by 2 secondary beats). The intervals between beats were constant at 0.493 s. Stimuli were presented using E-prime 2.0. Participants were asked to lie flat inside a two layer magnetically shielded room (MSR; Imedco) in front of a mirror projecting the visual instructions before each block and a fixation cross in the middle of the screen during the trials. The auditory stimuli were delivered via a STAX SRS-005 amplifier and SR-003 push pull electrostatic ear speakers outside the MSR, coupled by a vinyl tube to silicon earpieces to block any electromagnetic noise within the shielded room. Behavioral responses were collected using a LUMItouch photon control response box.

**Fig. 1.**
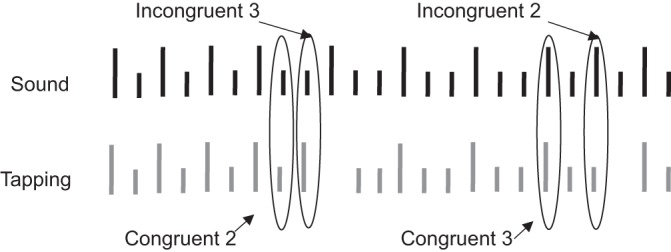
Meter-changing experiment. *Top*, drum beat times. Long lines indicate the primary beat, and short lines the secondary beat. *Bottom*, tapping performance. Long lines indicate taps with the 2nd finger, and short lines taps with the 3rd finger. Incongruent *event 3* and incongruent *event 2* represent the first incongruent tap after a change, whereas congruent *event 2* and congruent *event 3* represent the two forms of final congruent taps.

The subjects were asked to tap with their 2nd and 3rd fingers according to the beat. The 2nd finger was used for the primary beat and the 3rd finger for the secondary beats. The meter (2 or 3) was flipped at random, and the subjects were asked to follow the changes. In the experiment, subjects also heard nonchanging meters, tapped and heard nonchanging meters, or tapped with no auditory time cue. The entire duration of the recording was ∼35 min. This report only analyzes the condition where the subjects heard randomly changing meters and were asked to change their tapping to the beat. Figure 1 illustrates a short stretch of such behavior.

### Recording

Brain activity was recorded with an MEG (Magnes3600; 4D Neuroimaging) with 248 magnetometers and 23 reference magneto- and gradiometers, sampled at 1,017.25 Hz, and filtered between 0.1 and 400 Hz. Because an earlier version of our source localization software could not handle large files, the data from the first of the 9 subjects were sampled at 508.625 Hz and filtered between 0.1 and 200 Hz to reduce the file size. In addition to the magnetic fields, the following signals were recorded: the times of beat onset, the beat sound, tapping by each subject, the cycle-by-cycle times of the mains line power, and the *X*, *Y*, and *Z* acceleration of the MEG gantry.

### Anatomy

Head position indicators using five coils attached to the scalp provided exact information on the position of the head relative to the sensor array before and after the measurement. Coil positions were determined in relation to external anatomical landmarks. The head shape and coil position were digitized using a Pollhemus FASTTRAK digitizer.

Anatomical (T1 weighted) oblique slices were acquired along the anterior commissure-posterior commissure plane using a GE 3T scanner located at the Wohl Institute for Advanced Imaging at the Tel Aviv Sourasky Medical Center. The functional MEG data and the anatomical MRI data were coregistered using three anatomical landmarks (left preauricular, right preauricular, and nasion) and were manually fine-tuned to fit each subject's digitized head shape using AFNI (Analysis of Functional Neuro Images; Cox 1996).

### Data Processing

The MEG signal was cleaned of power line, acceleration, and heartbeat artifacts without affecting the rank of the data (Tal and Abeles 2013). In brief, the timing of each mains cycle was recorded in parallel to the MEG. For each MEG channel, the average signal around these marks was computed and subtracted cycle by cycle. Similarly, the mean heartbeat artifact was subtracted after detection of QRS peaks of the heartbeat artifact from the MEG channels. Calculations assumed a linear transformation between the gantry accelerations and the acceleration artifacts in the MEG. Thus the acceleration artifacts were cleaned in the frequency domain and then converted back to the time domain.

Source activities were reconstructed for 700 points evenly distributed over the cortical hull (see Fig. 3*A*). We chose these points to reconstruct the cortical signals from the entire cortex with a spatial resolution that was appropriate to the spatial resolution of MEG. The mean distance between neighboring points was 0.66 cm (SD 0.053 cm). If needed to better determine the type of area in which an event occurred, the coordinates of these points were converted to Talairach coordinates using the FreeSurfer image analysis suite (Fischl 2012). Supplemental Table S1 specifies the anatomical location of each of the points, their coordinates, and the total area of the anatomical region. (Supplemental material for this article is available on the *Journal of Neurophysiology* website.) We found significant correlation between the area of the region and the number of points selected in this region (*r* = 0.93), indicating that our points were approximately evenly distributed over the cortex. The amplitudes of the current dipoles at these 700 POI were evaluated. The evaluation method was similar to that described by Lots et al. (2013). In brief, we used simultaneous *N*-source reconstruction based on simultaneous synthetic aperture magnetometry (SAM; Robinson and Vrba 1999), which uses the covariance between the MEG measurements to reduce the effect of correlations among remote sources on the estimate. In this study we used the covariance matrix for the MEG data filtered at 15–30 Hz. Using higher frequencies provides better spatial localization, but higher frequencies have a lower signal-to-noise ratio (SNR). Beta-range frequencies appeared to be a good compromise between these effects. We further improved the localization of the source current by engulfing each POI on the hull by a cube with an edge length of 2 cm. Each of these cubes was rotated in 27 directions around its cardinal axes. For each cube, the current dipoles were simultaneously evaluated for all 8 + 1 points. Simultaneous *N*-source reconstruction by SAM is aimed at generating minimal correlations between the *N* points, thus providing a better estimate of the local activity at each point. Selecting the cube orientation with minimal correlation between the center and all eight corners enabled us to obtain the best estimate of the local currents at the center. We termed this evaluated current the “cortical current dipole” (CCD). In this way, 700 CCDs were obtained, each spanning the entire recording period.

One problem that arises when evaluating source amplitudes is that sources farther from the MEG sensors are artificially evaluated as having larger amplitudes. To avoid this amplitude bias, all CCDs were normalized to have zero mean and a variance of 1. Thus valid comparisons could only be made for the same CCD at different times.

We hypothesized that because input to the cortex is excitatory and most local cortical connections are excitatory, a new process in a region should start by a brief transient of increased activity. Support for this claim comes from the finding that such brief activity undulations can be used to discriminate five different mental activities (Abeles 2014). However, we needed a template for detecting such transients. This was obtained by finding the principal components (PC) for a sample of a few tens of such transients that were collected manually. Principal component analysis of these transients showed that a single PC covers more than 95% of the variance. A constant piece was added before this PC to facilitate detection of the first transient when a sequence of such transients followed each other. This was used to automatically search for such transients. In this search, the fit of the data was considered to be the signal, and the error between the data and the fit as the noise. The ratio (SNR) showed sharp peaks with varied amplitudes. Peaks that exceeded a chosen threshold were considered as points of optimal match to the template. This method is described elsewhere in detail (Abeles and Goldstein 1977). The mean signal around these detections was used as an optimal template for further detection of transients. A separate optimal template was defined for each of the 700 CCDs. Our hypothesis was that these transients indicate the beginning of a new neuronal process. Since mental processes such as syllable identification proceed at a rate of 2–3 per second, the threshold SNR for detection was set individually for each subject such that it yielded a rate of 2–3 transients per CCD per second. Occasionally, a huge spikelike wave was detected with this threshold. We considered these spikes to be outliers and rejected them. We defined the accepted transients as mini ERs.

Outlier rejection took place as follows: *1*) Let *X* be the list of peaks of the mini ERs squared. *2*) Compute the median and MAD (median of the average difference from the median) of *X*. *3*) Reject all points in *X* that are more than 7 MADs away from the median. This removed between 0.5% and 2% of the transients.

The detected mini ERs point up or down. The sign has little meaning since a dipole pointing up with a positive current is identical to a dipole pointing down with a negative current. In addition, because the localizing activity of the MEG is poor (∼2 cm), activity in one bank of a nearby sulcus may have the opposite sign than activity in the other bank. Therefore, in all computations, the peak of the mini ER was inverted if it was positive.

Some of the POIs were on the ventral surface of the brain, which is far from the sensors, and the space resolution there can be poor. To be on the safe side, when testing for time precision, we eliminated the CCDs of points in the ventral aspects of the brain. This procedure left us with 545–574 points for each subject. The text specifies which results were based on all 700 POIs and which were based on the reduced set.

Once the mini ERs were identified, we used these as the event times of a point process. Hence we converted the MEG recordings into 700 (or ∼565) parallel point processes recorded from 700 (or ∼565) points on the brain hull. We use the term “mini ERs” for both the events themselves and for the stream of times at which they occurred, but interpretation is clear from the context. Repeating patterns of mini ERs were searched for by implementing the algorithm described by Abeles and Gerstein (1988). In brief, the algorithm imitates the following idea put forward by G. L. Gerstein. Consider a representation of the point processes as a punched paper tape with time represented along the tape and a hole punch whenever an event occurs. If there are 700 parallel processes, there are 700 parallel lines of holes. Now, consider another copy of this tape. The two copies are shifted relative to each other. Wherever a pattern of events repeats, the representing holes will overlap. This detects all repeating patterns. If, for example, a pattern repeats three times, repetitions are detected when the first occurrence overlaps the second, when the second overlaps the third, and when the first overlaps the third. After detection of repetitions, the list of all repeating patterns is linted to be sure that each particular occurrence of a pattern is counted only once. The locations of points contributing to patterns were distributed all over the hull, at approximately equal density.

The search for repeating patterns was restricted to the time following the two types of congruent beats and the incongruent beats. Time sections from 10 to 493 ms after the beats were used. In the experiments there were about 90 changes of meter, so both patterns associated with congruent and with incongruent beats were found in about 45 s of activity. Patterns that repeated two or more times and included five or more mini ERs were searched separately for the data following the congruent beats and the incongruent beats.

**Fig. 2.**
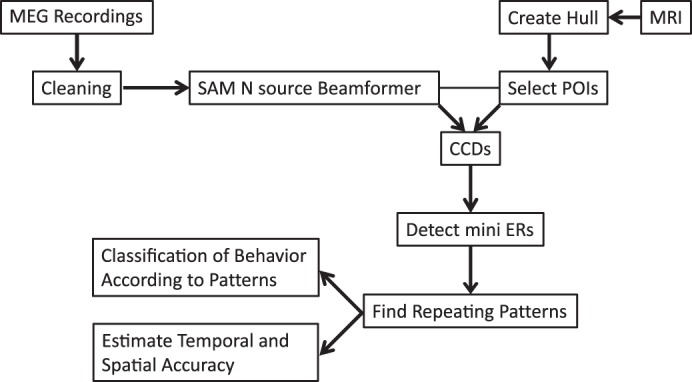
Analysis flow of the magnetoencephalographic (MEG) and MRI data. After cleaning, we applied a source localization procedure on selected points of interest (POIs) to generate cortical current dipoles (CCDs). We then detected repeating patterns in the mini evoked responses (ERs), estimated their spatiotemporal accuracy, and used these patterns for classification of the meter changes.

When we searched for pattern components that were specific to behavior, the occurrences of all possible pairs of events (mini ERs in *positions A* and *B* with a given interval Δ) in the two sets of patterns were examined. Pairs that repeated many times in patterns of the congruent beats and in none or very few patterns of the incongruent beats were considered as specific to the congruent beats and vice versa for the incongruent beats. Such pairs were called “specific” only if the probability of the difference in number of occurrences under the null hypothesis of no difference was below 1/1,000,000. These specific pairs were used as a seed for finding specific triplets, where the probability of the difference in number of occurrences by chance had to be below 1/1,000,000, as well. Figure 2 illustrates the analysis flow of the functional MEG data and the anatomical MRI data.

### Simulations

To verify that the temporal accuracy and the complexity of the detected patterns were not a consequence of our analysis method, we used two different simulations. The first simulation was based on random sets of numbers, and the second was based on segments from real MEG recordings.

#### Random numbers simulation.

We simulated the neuronal responses using truly random numbers generated from physical measurements (Kanter et al. 2010). The purpose of this simulation was to test whether the temporal accuracy of the patterns found in the real MEG data could also be found in random numbers. The random numbers were filtered at 8–60 Hz and normalized to have zero mean and a variance of 1. Mini ERs were found in the filtered data according to the same algorithm described above. We then divided these data into 700 channels, each containing 90 segments of 0.5 s, and searched for repeating patterns using the same procedure as employed on the real MEG data.

#### MEG data simulation.

In the above simulation we used random numbers. However, in the real data there were also responses embedded in the CCD. To account for this, we picked 90 random segments from the experiment. Having constructed null data in which any repetitions of mini ERs could only have occurred by chance, we then repeated the above analysis to evaluate the null distribution of their temporal pattern statistics. To simulate condition-specific effects, we added the mean congruent or incongruent responses to each piece of scrambled data. These are referred to below as the pseudo congruent and incongruent beats. All the pieces were then concatenated, and mini ERs were searched for. Since the SNR was set to yield a rate of 2–3 mini ERs per second over the entire recording period, we used the same SNR value (SNR = 3.5). The resulting point processes were then split between the pseudo congruent and pseudo incongruent (scrambled) beats. Searches for specific pairs and triplets of events were carried out both for the real data and for 100 random scrambles and generated pseudo responses in the same way. This procedure allowed us to create the null distribution of patterns specific to a given cognitive behavior and compare it with the number of specific patterns detected in the real data.

## RESULTS

### Brain Responses

For comparison purposes, the behaviors were grouped into two comparable sets. The first set consisted of cases in which the subject's tapping was incongruent with the beat; that is, the subject tapped a primary beat and heard a secondary beat (after the meter changed from 2 to 3; see Fig. 1, “Incongruent 3”) or tapped a secondary beat but heard a primary beat (after a change from 3 to 2; see Fig. 1, “Incongruent 2”). Note that for all subjects reported here, tapping was entrained to the beats so that tapping occurred before the beat was heard, an anticipatory action that is well-documented (Repp 2005; Woodrow 1932). After realizing the meter had changed, most subjects did not tap the next beat and only later resumed correct tapping.

The second set consisted of the last times at which tapping and beats were congruent. In other words, in these cases the subjects tapped a primary beat and heard a primary beat (see Fig. 1, “Congruent 2”) or tapped a secondary beat and heard a secondary beat (see Fig. 1, “Congruent 3”), but on the following tap of the same kind there was an error. Thus there were equal numbers of cases in the congruent and incongruent sets. Both contained activities related to tapping with the 2nd finger and with the 3rd finger and hearing a primary and a secondary beat. By including responses with both fingers (and evoked by a primary or secondary beat), we were able to focus on the cognitive processing indexed by the mini ERs, or in other words, coherent fluctuations that could not be explained by the sensory or motor aspects of neuronal processing.

**Fig. 3.**
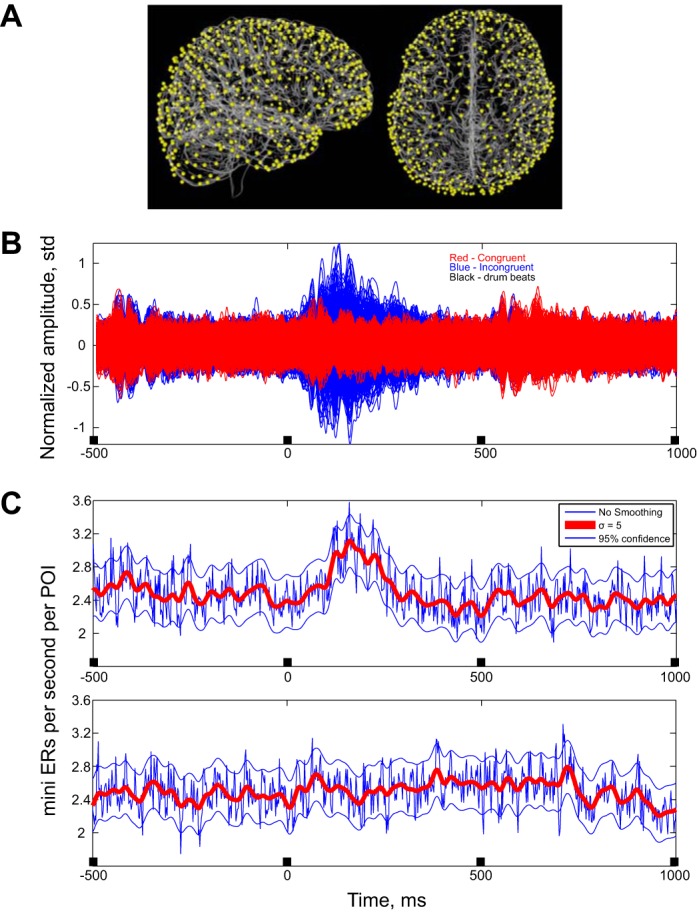
Average activities. *A*: anatomical locations of POIs. The cortical surface of a single subject is shown in sagittal and axial views. The surface was created using FreeSurfer and overlaid by the locations of the POIs contributing to the pattern density. *B*: CCDs in the congruent and incongruent cases. All the CCDs were filtered at 8–60 Hz and then averaged around the beat. Amplitudes of the CCDs were normalized to have 0 mean and a variance of 1. Blue (amplitude around the incongruent beat) averages are large even compared with the ongoing background activity. Red traces indicate amplitude around the last congruent beat. The red traces are plotted over the blue ones. *C*: mean rate for the mini ERs on all channels for the incongruent case (*top*) and for the congruent case (*bottom*). Blue traces indicate mean rate with a maximal time resolution (∼2 ms). Red traces indicate rate after smoothing with a Gaussian bell with a standard deviation of 5 ms. Black marks on the *X*-axis indicate the drum beat times.

Figure 3 shows the anatomical locations of the mini ERs for one subject and a superposition of all 700 mean CCDs for the incongruent events (blue) and the congruent events (red). At *time 0* the subject heard the beat, taking into account the time it took for the sound to travel from the earphones through the conducting tubes to the eardrums. Times −493, +493, and 986 ms indicate when the preceding and following beats were heard.

Clearly, in multiple CCDs there was a much greater response to incongruent beats than to congruent ones. From the perspective of classical event-related potential studies, the more exuberant responses during the incongruent condition may reflect the well-known oddball effects on electrophysiological responses. These have often been interpreted in terms of predictive coding and a greater expression of prediction error during unpredicted incongruent (stimulus) processing. In the present study, we found the same effect but expressed in terms of mini ERs. The rate of mini ERs increased following an incongruent beat (Fig. 3*C*, *top*). Therefore, the subsequent analyses concentrate on times 10 to 490 ms after incongruent beats.

### Mini ERs

**Fig. 4.**
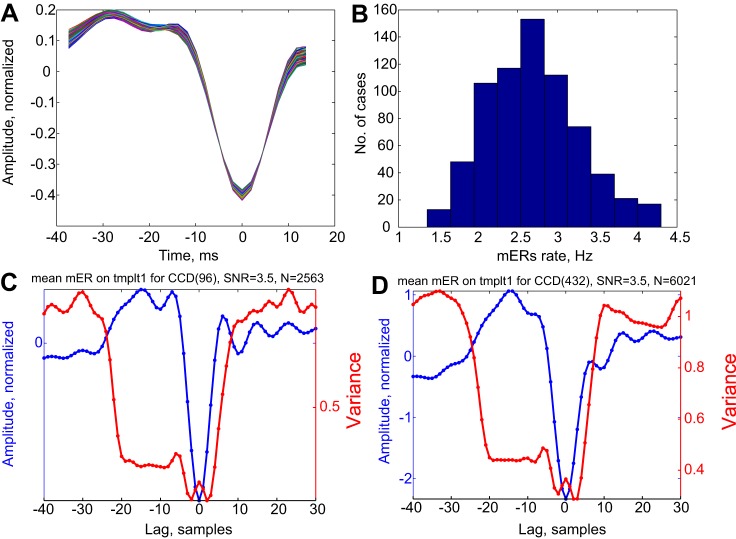
Properties of mini ERs. *A*: superposition of 700 templates to detect mini ERs. Each template was normalized to have 0 mean and a norm of 1. Colors were added to facilitate discrimination between traces. *B*: distribution of mini ER rates across the different CCDs. *C* and *D*: CCDs were filtered at 8 to 60 Hz, and the mean wave shape around the time of mini ER detection was computed. Examples of mean mini ER and the variance around them are shown. The variance corresponds to the dispersion of current source density about the mean as a function of time after time-locking to mini ERs. Note that in contrast to what is usually observed for averaged responses, the variance drops precipitously for the duration of the template (lag: −21 to 5 samples.). Note that the variance is ∼1, and therefore the standard error of the mean is less than 0.02 for both *C* and *D*, whereas the scale for the mean spans ∼3 standard deviations. Plotting such a band around the mean would not leave room for the mean itself to be seen. The sampling rate was 508.36 Hz, so the interval between samples was ∼2 ms. mER, mini evoked responses; SNR, signal-to-noise ratio; templt1, template.

**Fig. 5.**
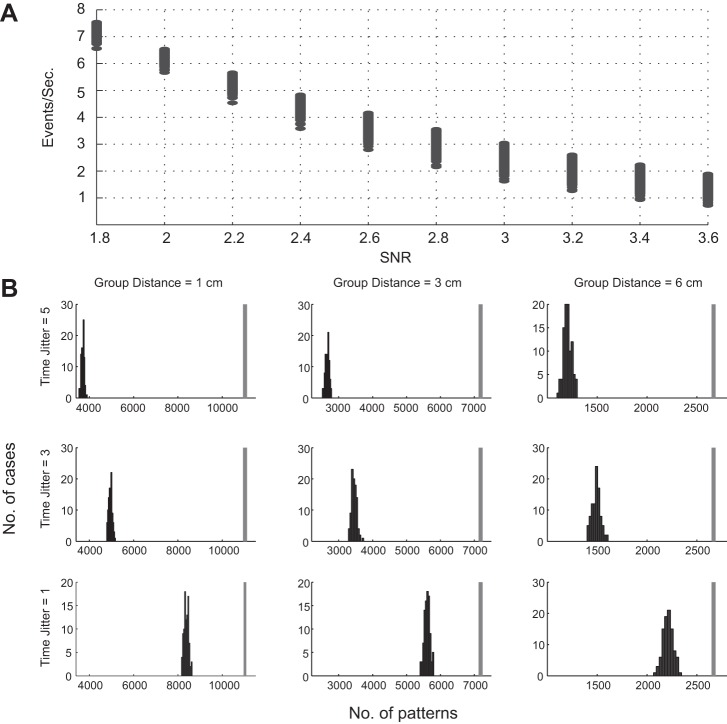
Number of events and patterns for 1 subject. *A*: number of mini ERs as a function of SNR threshold for detection. Each point represents 1 channel. The SNR value that yielded ∼2–3 events per second for each channel in this subject was 3. *B*: histograms indicate the total number of patterns found in 100 teeterings. Vertical gray bar indicates the number of patterns in the real data. Both the real number and the teetered numbers are based on reducing the events so that any synchronous group could be split into groups with a diameter <1, 3, or 6 cm and intergroup distances >1, 3, or 6 cm, respectively. *Top* row, teetering within ±5 samples; *middle* row, teetering within ±3 samples; *bottom* row, teetering within ±1 samples.

Templates for detecting mini ERs were found in a two-step process. As a first step, we collected all the samples that fit the available template for each channel (Abeles 2014). The detection threshold was set individually for each subject to an SNR value between 3 and 3.5 to provide around 2.5 detections per second (see Fig. 5*A*). As a second step, the mean wave shape over all detections was used as a template for further mini ER detection using the same SNR. Figure 4*A* shows that for all CCD channels, the templates were almost identical. However, the number of mini ERs varied across the different channels. The distribution of mini ER rates is shown in Fig. 4*B*. The mean mini ER for CCDs 96 (with the least number of mini ERs) and 432 (with the highest number) are illustrated in Fig. 4, *C* and *D*. Also shown is the variance around this mean. Note that the variance is plotted on a different *Y*-axis, because if the standard errors around the mean amplitude were drawn, they would be too small to evaluate by visual inspection. The variance falls two to three times for the entire duration of the template (−42 to 10 ms) but may have a small secondary peak near *time 0*, showing variability in the amplitude of the sharp peak. This substantial reduction in the variance means that at the time of the mini ERs, there was a major reorganization of the activity at that location. This is not unexpected, since mini ERs were only detected when the SNR was >3. However, if this was an unreasonably high SNR threshold, there should not be many such cases in each channel.

The rate of mini ERs was modulated in the incongruent condition. Figure 3*C* shows that the rate increased around 90–300 ms after the drum beat was heard. The large responses seen on many CCDS tend to mask the ability to detect mini ERs. Thus the rate increase seen in Fig. 3*C* is probably an underestimate of the true rate. All further analyses below relate to mini ERs observed consecutive to the incongruent beats.

### Precise Time Sequences of Mini ERs

We searched for precise sequences of mini ERs that repeated themselves at least twice. The maximal duration of the sequence was limited to 40 ms, and initially only patterns composed of 4 or more events were retained. For example, in one experimental day, when looking for patterns in the data set following an incongruent beat, we found 15,294 repeating patterns composed of 5 mini ERs each with exactly the same delays between successive mini ERs. Similarly, there were 3,145 patterns with 6 mini ERs, 692 with 7, etc. The most complex was a pattern with 14 mini ERs. We did not break down complex patterns into all their possible subpatterns (e.g., in a pattern with 14 events, there could be 2,002 different patterns with 5 events). However, all 19,329 patterns only repeated twice!

### Time Precision of Repeating Patterns

Time precision can be estimated by finding the effect of teetering event times randomly within a time window *W* (Amarasingham et al. 2012; Date et al. 1998; Harrison et al. 2014; Harrison and Geman 2009; Shmiel et al. 2006). The source localization ability of the MEG is limited, so mini ERs occurring among a few adjacent POIs may show up on all or a few of them. Thus, instead of individually teetering every mini ER time or all the coincident mini ERs together, we can do so in groups. The diameter of the groups (*D*) depends on the spatial accuracy of the estimation of the CCDs which, in turn, depends on the distance of the POI from the MEG sensor. To overcome this complication, we excluded POIs on the ventral aspect of the brain from the data. This left us with 545–574 POIs.

Teetering was then done as follows. We divided the measurement time into segments each spanning three samples. In each such temporal segment there were 11 .3 ± 7.3 mini ERs over all POIs. We looked at the positions at which these near-synchronous mini ERs occurred. These positions were divided into groups of diameter *D* such that within each group, the distances between any two positions did not exceed *D*. In addition, we made sure that the distance between a foreign pair, i.e., one from one group and the other from another, was greater than *D*. If not, we deleted the offending mini ER from the list of mini ERs in that time slice, and this was iterated until the distance between groups was greater than *D*. Note that the number of remaining events was smaller as *D* became larger, and therefore, a smaller number of repeating patterns was found for larger *D*.

To find the time precision of the repeating patterns, we teetered the mini ER times within a window *W*, teetering together mini ERs that were coincident within [−1, 1] samples and whose locations were within the diameter (*D*) of 1, 3, or 6 cm. We then searched for repeating patterns in the teetered data. This was repeated 100 times, and the distribution of the number of patterns was computed. The rationale was that if the actual number of patterns was not within this distribution, the time accuracy was better than *W* with a probability of less than 1%. Figure 5 illustrates this for one subject.

In each column of Fig. 5*B*, the number of patterns in the unteetered data stayed the same, but as *W* became smaller, the histogram moved toward the actual number. However, even for the smallest possible teetering (±1 sample) and the largest group diameter (6 cm), the number of repeating patterns in the real data was significantly greater than that of the teetered data. Since in this example the data were sampled at ∼1,000 Hz, ±1 sample spans almost 3 ms. In all subjects, the number of repeating patterns in the teetered data never reached the number of patterns in the real data.

To estimate the differences of parameters between the real and the jittered data for all subjects, we used Bayesian estimation as suggested by Kruschke (2013). This method provides precise estimates of statistical power and handles outliers, small sample size, and nonnormality of the data. Since the dynamics of cognitive processes is likely to be extremely specific to every individual, pulling together all subjects as though they are samples from a homogenous population is bound to have very low statistical power. Therefore, our group analysis was conducted as follows. For each subject, we calculated the ratio between the number of patterns found in the real data and the number of patterns found after the first attempt to jitter the events within ±1 sample. As a control group, for each subject, we calculated the ratio between the number of patterns found in the first and second attempts to jitter the event times. Using this procedure, we were able to compare the distributions using Bayesian estimation and quantify the difference between the real and the jittered data at the group level.

**Fig. 6.**
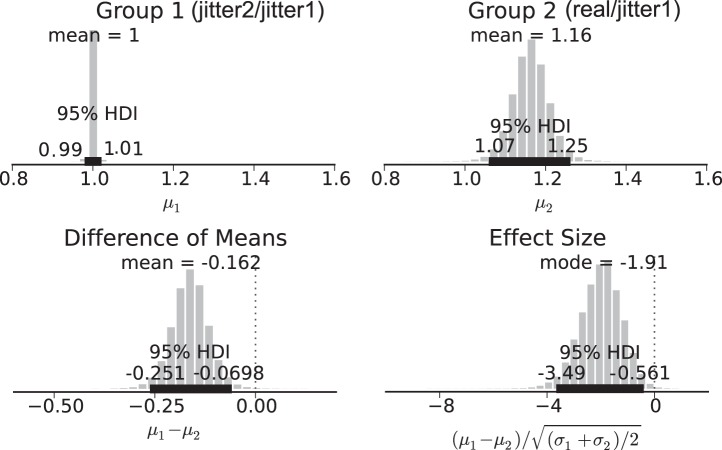
Bayesian estimation for number of patterns. *Top* row, mean ratio between the number of patterns in 2 successive jitters (*left*) and between the real data and the jittered data (*right*). *Bottom* row, difference in means (*left*) and effect size (*right*) distributions. From these distributions we can take the mean credible value as our difference and the 95% highest density interval (HDI) as the range where the actual difference is with 95% credibility.

Figure 6 shows the probability distribution of the two groups (*top*) and the effect size and difference in means distributions (*bottom*). Since the highest density interval (HDI) of the difference between the groups is greater than 0, there is a credible difference between the number of patterns found in the real and the jittered data. Similarly, a significant difference in the standard deviations was found.

### Random Numbers Simulation

**Fig. 7.**
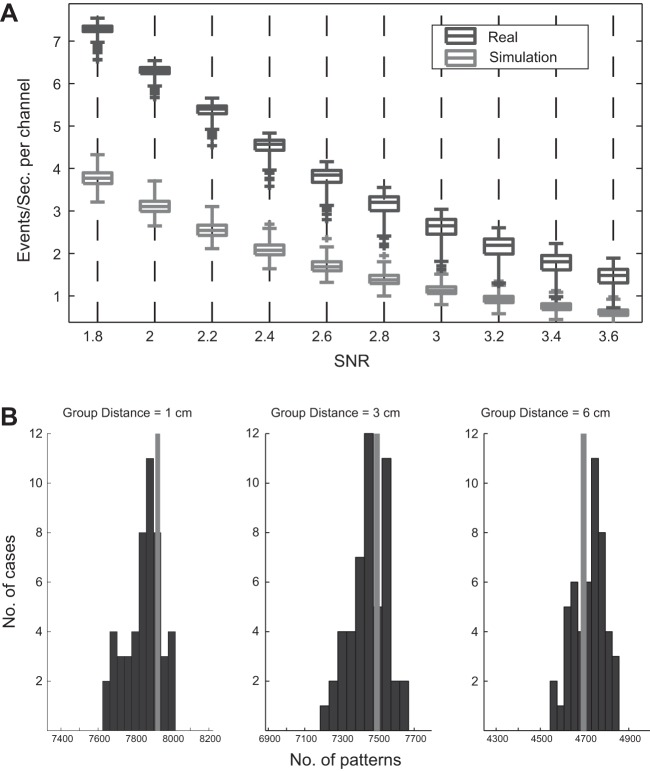
Number of events and patterns for the simulated data. *A*: number of mini ERs as a function of the SNR threshold for detection in the real MEG data for a representative subject (black) and in the simulation (gray). The boxes show the 1st and 3rd quartiles of the mini ER rate across channels (with the median, minimum, and maximum). Outliers (over ±3 SD) are marked by a plus sign (+). *B*: histograms indicate the total number of patterns found in 50 teeterings. Gray bar indicates the number of patterns in the data before teetering. Both the real numbers and the teetered numbers are based on reducing the events so that any synchronous group could be split into groups with a diameter <1, 3, or 6 cm and intergroup distances >1, 3, or 6 cm, respectively. In the simulated data, teetering within ±1 samples did not affect the number of patterns in the simulated data.

In the simulated data we used SNR = 2.2 as the threshold for detection of mini ERs, which provided two to three events per second for each channel (Fig. 7*A*). This meant that to find the same rate of mini ERs in the simulated data, we had to decrease our SNR threshold compared with the real MEG data. In the simulated data, even though we had a similar number of events, the number of repeating patterns and their maximal complexity were reduced to 7,930 and 7, respectively. To find the time precision of the repeating patterns in the simulated data, we used the teetering idea with a time window of ±1 ms and a group diameter *D* of 1, 3, and 6 cm. The distance between channels in the simulated data was taken from the distance measured in one of the subjects.

Figure 7*B* shows the distribution of number of patterns in the teetered data for 50 permutations. When the events in the simulated data were teetered, the actual number of patterns fell within the distribution of the teetered data. This result indicates that unlike the real MEG data, the number of patterns in the simulated data was not affected by the teetering process in any of the group diameters. That is, there was no time precision in the patterns derived from the random data.

### Behavioral Specificity of Patterns

#### MEG data simulation.

**Fig. 8.**
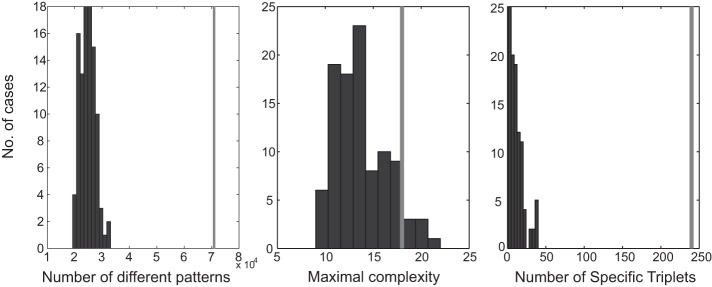
Distribution of parameters for the scrambled incongruent beats. Black bars represent the distribution in 100 scrambled pieces. Gray bars indicate the value in the real data. *Left*, the number of different repeating patterns starting with quintuplets. *Middle*, the length of the patterns with the maximal number of events. *Right*, the number of triplets that repeated many times in congruent beats and never or only a few times in the incongruent beats, where the difference had a surprise of 6 or more.

Figure 8 summarizes the results of the simulation by using random time segments from the real MEG data. Clearly, the maximal complexity of repeating patterns did not differ from that of the real data. However, for all other parameters there was a huge difference. Note that the number of patterns and number of specific triplets are not independent measures. Nevertheless, the enormous differences between the real and scrambled data show that in the real data there are repeating patterns (and subpatterns) that are strongly associated with behavior.

#### Classification of behavior.

We then tested whether repeating patterns could be used to differentiate between the two behaviors (congruent and incongruent beats). This called for splitting the data into training and test sets. The time accuracy of the mini ERs was relaxed so that a jitter of ±1 sample was allowed.

Many thousands of such repeating patterns were found. Due to computational time limits, we restricted our attention to quadruplets. We started with quadruplets repeating two or more times. There were ∼11,000 such quadruplets repeating 2 to 6 times.

The data were split into training and test sets, each containing equal numbers of trials for both the congruent and incongruent beats. Quadruplets that repeated at least twice in one training set and not even once in the other were considered specific to that behavior. In this way we obtained ∼2,500 specific quadruplets for the congruent behavior and ∼2,000 for the incongruent behavior. For each test set we counted how many patterns considered specific to one (or the other) behavior were found. The test set was assigned to the behavior for which a majority of specific patterns were found.

A different random split into training and test sets was then carried out, and the whole procedure was repeated. The split was repeated 50 times so that we could estimate the probability that a test set that came from one behavior would be assigned to this behavior or the other.

Table 1 shows the average classification results of 4 experimental days sampled at a rate of 1,017.25 Hz. Data from the congruent beats were assigned to congruent beats 89.5% of the time, whereas data from the incongruent beats were assigned to incongruent beats 89% of the time. This is a fairly good characterization. In four experimental data sets, the worst was 60% and the best was 98% correct characterizations.

**Table 1.** Ability of quadruplets to discriminate between the two behaviors

	Conditional Probabilities	
	Minimum Repeats ≥2	
Congruent		Incongruent
Congruent	0.895	0.105
Incongruent	0.11	0.89

## DISCUSSION

Our analysis was based on the working hypothesis that whenever a new type of process starts in a given cortical region, activity will initially increase and then be quenched by the local inhibition. Such transient increases will manifest themselves as mini ERs. This supposition derived from our earlier finding that it is possible to correctly discriminate among five types of mental processes via the density maps of mini ERs over the brain's hull (Abeles 2014). The aim of the present study was to examine the dynamics and organization of these mini ERs in space and time, with particular emphasis on the time precision of repeating patterns of activations. The main results show that repeated patterns of complexities from 3 to 22 events can be found in human neurophysiological data. Such patterns indicate that cortico-cortical interactions can be very temporally precise, and in fact better than 3 ms. The approach developed in the present work opens new vistas for studying the evolution of human cortico-cortical interactions during the performance of a cognitive task. It also calls for analysis of the mechanisms by which such precise patterns are generated.

In contrast to functional MRI studies, which give a quasi-static impression that a given region is involved in one process or another, the approach presented in this report captures the distribution and dynamics of involvement of multiple cortical areas on the timescale of a few milliseconds.

Cortico-cortical interactions in human electrophysiological recordings are usually assessed by using methods that measure correlations between time series, synchrony in different frequency bands, or phase interactions. In the current study we analyzed the MEG recordings by using methods adapted from animal electrophysiology that are suitable for point process analysis. To the best of our knowledge, this is the first attempt to identify such precise spatiotemporal patterns from MEG recordings in humans. Thus the issue of how these spatiotemporal patterns relate to findings obtained using existing methods is an open question that requires further work. Several studies have described the existence of precise spatiotemporal patterns in recordings from the cerebral cortex (Abeles 1982; Dayhoff and Gerstein 1983; Haalman and Vaadia 1998; Klemm and Sherry 1981; Kumbhani et al. 2007; Mackevicius et al. 2012; Markram et al. 1997). In human electrophysiological recordings, the focus of analysis is based on the presence of oscillations (Buzsáki 2006; Logothetis et al. 2001), the temporal structure of nested frequencies in scale-free dynamics (He et al. 2010), and cascades of activity described as neuronal avalanches (Shriki et al. 2013). The current analysis emphasizes the significance of the temporal accuracy of neuronal activity measured by MEG and its specificity to cognitive tasks.

The reader will notice that we needed to adopt a number of bespoke analyses and adjustable parameters that were set according to our physiological interpretation to characterize repetitions of point processes indexing mini ERs. The statistical analysis of these point processes (and patterns) is a difficult and contentious area that has a long history. In this sense, we present our results at a descriptive level while trying to quantify the spatiotemporal expression of systematic responses throughout the cortex. Our focus is on the quantitative results, as opposed to a categorical inference of significance. Having said that, all the results reported herein survived correction in relation to null distributions obtained by jittering or resampling and allowed us to classify the cognitive tasks according to specific patterns.

Our data are based on recordings from 9 subjects. This is not a large number. However, in all subjects we obtained the following results: *1*) the pattern accuracy was better than ±1 sample; *2*) the number of specific triplets was much higher compared with surrogate data derived from the MEG of the same subject; and *3*) the ability to discriminate between the congruent and incongruent beats in a test set on the basis of patterns detected in a training set was much better than chance. Even if our methods were completely wrong and the probability of success in each test was 0.5, the probability of succeeding in 9 of 9 trials would be better than 0.002. The probability of such successes in all three criteria mentioned above is miniscule. Thus the conclusion that precise patterns exist and that at least some of them and/or some parts within them are specific to the underlying cognitive process is unavoidable.

We extracted group mean values of the number of precise patterns by using Bayesian estimates of the differences between the real data and the jittered data (Kruschke 2013). When the data for each subject are examined, such parameters vary on an extremely wide range. The specifics of the spatiotemporal dynamics of a given cognitive process in each subject may depend on personality and past experience and therefore show large individual differences. Thus we compared the ratio between the number of patterns detected in the real data and the number of patterns detected in the jittered data of each subject. In all group diameters we found significant differences between the real and the jittered data for all subjects, indicating the importance of the exact timing of the mini ERs.

It could well be that selecting an SNR threshold of 3–3.5 resulted in the loss of many mini ERs or the inclusion of unrelated mini ERs in our data. We selected this threshold because it yielded, on average, ∼2.5 events per channel per second. This seemed to be an adequate rate for new mental processes in each location. However, the effect of the threshold on the results should be studied further.

When checking the validity of data, one typically uses random numbers that are generated by some algorithm. These may look random when low-order statistics are examined, but the rule by which they are generated may show up when high-order properties are examined. Therefore, we used a truly random bit stream generated by a physical process (Kanter et al. 2010). This bit stream was chunked to nonoverlapping 16-bit pieces that were considered a stream of random numbers. In sequences of random numbers, one can also find a number of sharp transients (pseudo mini ERs). However, to detect the same rate of mini ERs, we had to reduce the threshold for detection. When the SNR was set to 3 (as in the real data), the number of mini ERs in the random data was reduced 3-fold. Thus approximately one-third of the mini ERs in the real data could be chance events. The real mini ERs generated more repeating patterns than the pseudo mini ERs. The patterns of pseudo mini ERs showed no time precision (Fig. 7), whereas for the true patterns it was better than ±1 sample (Fig. 5). The true patterns showed good specificity to behavior (Table 1). This leads to the conclusion that a nonnegligible number of the mini ERs in the real data do represent specific brain processes.

When a single channel of real data was compared with the random number stream, two major differences were observable: *1*) the real data contained bursts of oscillations, and *2*) the real data also contained event-related activities. Our template for mini ER required that the sharp transient be preceded by a relatively flat trace. Thus, when an oscillating burst occurred, only the first wave would be detected as a mini ER. In our experiment, the subject listened to drum beats and tapped with one finger, or both, every ∼0.5 s most of the time. Therefore, event-related activities were superposed on ongoing activities most of the time. These ERs reduce the likelihood of finding mini ERs. This means that in the real data, there were actually more mini ERs, but some were masked either by bursts of oscillations or by large ERs. Although our analysis of patterns definitely shows that we did reveal brain activities through mini ERs, it should be recalled that a nonnegligible part may have been chance events and that a nonnegligible part was not detected by our algorithm. To overcome these issues, we ran another simulation using random segments from the MEG recordings and added the evoked responses to these segments as described in methods. The fact that we detected more triplet patterns in the real data that were specific to a certain behavior compared with the simulation suggests that these patterns indeed represent a specific cognitive process and are not biased by oscillations or the presence of evoked responses.

The search for mini ERs was motivated by the physiological nature of cortico-cortical and thalamo-cortical connections, which are excitatory, whereas inhibition is local in the cortex. Hence any increase in input to the cortex will initially yield increased local activity, which will then be restrained by the local inhibition. The balancing effect of inhibition may be immediate or may result in ringing oscillations that quench gradually. Our template tended to find the first wave in such ringing, but the small gamma-range oscillations following the mini ER, as shown in Fig. 4, *C* and *D*, indicate that some were indeed followed by such ringing.

The temporal precision of repeating patterns was assessed by teetering events randomly within different time windows and at different group diameters. The fact that teetering, even within ±1 ms, significantly reduced the number of repeating patterns found suggests that there is a significance in the precise timing of the mini ERs. On the other hand, such precise timing was not found in the simulations with random numbers, thus ruling out the possibility of bias in the analysis. This result reinforces the hypothesis that the cerebral cortex utilizes the time domain for processing.

Our initial interest in timing accuracy was motivated by the thought that cross-cortical interactions are mediated by binding among synfire chains (Abeles 1982; Abeles et al. 1993, 2004; Hayon et al. 2005). In such a scenario, early stages of a chain may evoke activity in remote cortical areas, whereas later stages may evoke activity in nearby regions. In this way, short delays in activation of remote sites would be generated. Although our results are consistent with the idea that elementary cognitive processes are carried by synfire chains and complex processes by binding among synfire chains in multiple regions, they by no means prove this conjecture. Other mechanisms may enable precisely repeating patterns, as well.

In the repeating spatiotemporal patterns of four or more events, only very rarely did patterns repeat more than twice. The maximal number of repetitions was seven. However, lower order patterns (triplets) could repeat many times. For example, in one of the subjects there were 187 triplets that repeated more than 4 times out of 134,620 that repeated more than once.

The cognitive processes of realizing that the meter had changed, recalling the new meter, and recalling the motor program for tapping according to the new meter repeated ∼90 times in our data. Why then were there so few repetitions of the same patterns following the same type of stimulus? Four types of shortcomings may have contributed to this outcome: *1*) we detected mini ERs on a trial-to-trial basis and therefore may have missed quite a number of them; *2*) a considerable portion of the mini ERs may have been chance events that interfered with our ability to observe repetitions; *3*) concomitant brain processes may have added irrelevant mini ERs that masked the repetition of the pattern specific to the process; and *4*) the same behavioral end result could be the outcome of several different forms of cortical processing, much like a multiprocessor computer can execute the same algorithm using different physical resources depending on the scheduler state, RAM, register allocation, etc. Regardless, our analysis shows that even in the presence of such limitations, we were able to detect patterns that were specific to behavior.

## GRANTS

This research was supported in part by the I-CORE Program of the Planning and Budgeting Committee and The Israel Science Foundation Grant 51/11.

## DISCLOSURES

No conflicts of interest, financial or otherwise, are declared by the authors.

## AUTHOR CONTRIBUTIONS

I.T. and M.A. conception and design of research; I.T. performed experiments; I.T. and M.A. analyzed data; I.T. and M.A. interpreted results of experiments; I.T. and M.A. prepared figures; I.T. and M.A. drafted manuscript; I.T. and M.A. edited and revised manuscript; I.T. and M.A. approved final version of manuscript.

## Supplementary Material

Table S1
